# National cross-sectional study on cost consciousness, cost accuracy, and national medical waste reduction initiative knowledge among pediatric hospitalists in the United States

**DOI:** 10.1371/journal.pone.0284912

**Published:** 2023-04-24

**Authors:** Benjamin C. Lee, Matt Hall, Ladan Agharokh, Andrew G. Yu, Kavita Parikh, Samir S. Shah

**Affiliations:** 1 Division of Hospital Medicine, Children’s Health Dallas and Department of Pediatrics, University of Texas Southwestern Medical Center, Dallas, TX, United States of America; 2 Children’s Hospital Association, Lenexa, KS, United States of America; 3 Division of Hospital Medicine, Children’s National Hospital and Department of Pediatrics, George Washington University School of Medicine, Washington, DC, United States of America; 4 Division of Hospital Medicine, Cincinnati Children’s Hospital Medical Center and Department of Pediatrics, University of Cincinnati College of Medicine, Cincinnati, OH, United States of America; Iuliu Hațieganu University of Medicine and Pharmacy: Universitatea de Medicina si Farmacie Iuliu Hatieganu, ROMANIA

## Abstract

**Background/objective:**

Despite initiatives to reduce waste and spending, there is a gap in physician knowledge regarding the cost of commonly ordered items. We examined the relationship between pediatric hospitalists’ knowledge of national medical waste reduction initiatives, self-reported level of cost-consciousness (the degree in which cost affects practice), and cost accuracy (how close an estimate is to its hospital cost) at a national level.

**Methods:**

This cross-sectional study used a national, online survey sent to hospitalists at 49 children’s hospitals to assess their knowledge of national medical waste reduction initiatives, self-reported cost consciousness, and cost estimates for commonly ordered laboratory studies, medications, and imaging studies. Actual unit costs for each hospital were obtained from the Pediatric Health Information System (PHIS). Cost accuracy was calculated as the percent difference between each respondent’s estimate and unit costs, using cost-charge ratios (CCR).

**Results:**

The hospitalist response rate was 17.7% (327/1850), representing 40 hospitals. Overall, 33.1% of respondents had no knowledge of national medical waste reduction initiatives and 24.3% had no knowledge of local hospital costs. There was no significant relationship between cost accuracy and knowledge of national medical waste reduction initiatives or high self-reported cost consciousness. Hospitalists with the highest self-reported cost consciousness were the least accurate in estimating costs for commonly ordered laboratory studies, medications, or imaging studies. Respondents overestimated the cost of all items with the largest percent difference with medications. Hospitalists practicing over 15 years had the highest cost accuracy.

**Conclusions:**

A large proportion of pediatric hospitalists lack knowledge on national waste reduction initiatives. Improving the cost-accuracy of pediatric hospitalists may not reduce health care costs as they overestimated many hospital costs. Median unit cost lists could be a resource for educating medical students and residents about health care costs.

## Introduction

Health care spending accounts for 19.7% of United States gross domestic product [1], with hospital expenditures accounting for $1.3 trillion, or nearly one-third of all health care expenditures. The Institute of Medicine estimated that 30% of health spending was wasteful and they advocated for better care at lower costs [2]. Pediatric health care spending has dramatically increased from $150 billion in 1996 to $235 billion in 2013 [3, 4]. National medical waste reduction initiatives such as the American Board of Internal Medicine Foundation’s Choosing Wisely® campaign and the Pediatric Respiratory Illness Measurement System (PRIMES) aim to reduce unnecessary medical tests, treatments, and procedures through consensus recommendations [5, 6]. The Accreditation Council for Graduate Medical Education stresses the importance of “incorporating considerations of value, cost awareness, delivery and payment, and risk-benefit analysis”” and “understanding health care finances” during resident training [7]. Physicians believe that they have a major responsibility to address health care costs [8] and that containing costs is the responsibility of every physician [9]. Despite these charges, a majority of residents, fellows, and attendings (physicians who have completed medical training) do not know the cost of commonly ordered items [10–13].

Current pediatric studies examining cost consciousness or cost accuracy are at single institutions or at a few sites and involve specialties outside of hospital medicine [10, 11, 14, 15]. Studies have explored barriers to implementing Choosing Wisely® recommendations [16], as well as initiatives incorporating them into training programs [17]. In high-value care, medical benefits outweigh the cost of interventions. We hypothesized that knowing the cost of commonly ordered laboratory studies, medications, and imaging studies may help physicians improve high-value care. We also sought to determine whether pediatric hospitalists’ knowledge of national medical waste reduction initiatives and self-reported level of cost-consciousness (the degree in which cost affects practice) were associated with knowledge of costs and cost accuracy (how close an estimate was to its hospital cost) for commonly ordered items at a national level.

## Methods

This is a cross-sectional study utilizing an online survey and the Pediatric Health Information System (PHIS; Children’s Hospital Association, Lenexa, KS).

### Survey

A 31-question survey was created using Research Electronic Data Capture (REDCap; Vanderbilt University, 2021). Survey topics were determined from national medical waste reduction initiatives [6, 18], previous literature on cost consciousness [11], and author consensus. Common laboratory studies, medications, and imaging studies were selected from a list of the top 100 ordered items for each category, according to PHIS data, and focused on those linked to national medical waste reduction initiatives such as Choosing Wisely® or Pediatric Respiratory Illness Measurement System (PRIMES). The survey was piloted by several co-authors and revisions were made to the survey wording and number of selections.

Since there was not an all-inclusive contact list for pediatric hospitalists (attendings who spend a majority of their time caring for hospitalized patients), an email invitation with a general survey link was sent to pediatric inpatient (Hospital Medicine or General Pediatrics) division directors or division research leaders at all 49 hospitals that contribute data to PHIS. A reminder was sent every 2 weeks (3 total reminders) and the survey duration was 8 weeks, from April 2021 to May 2021. Division directors were identified via hospital or medical school websites, calling division offices, or previous relationships with authors. The directors were asked to forward the survey link to pediatric hospitalists. The UT Southwestern Human Research Protection Program (HRPP) determined that the study does not meet the definition of research under 45 CFR46.102, and therefore did not require IRB approval or oversight. Participants were not required to provide informed consent, but completion of the survey acknowledged participation in the study. Responses were anonymously analyzed. There were no financial incentives for respondents. The response rates were calculated using American Association of Public Opinion Research response rate definitions 5 and 6 [19].

Demographic information was collected about each participant’s gender, years in practice, current year of training, and primary location of clinical duties. Participants reported their familiarity with national medical waste reduction initiatives and how frequently cost consciousness affected their ordering of common laboratory studies, medications, and imaging studies in a typical week, using a Likert-type scale (S1 File). Lastly, participants assigned a numerical dollar estimate to the cost of common laboratory studies, medications, and imaging studies at their hospital.

### PHIS data

PHIS is a comparative administrative database which includes clinical and resource utilization data for inpatient, ambulatory surgery, emergency medicine, and observation unit patient encounters for 49 United States children’s hospitals [20]. Median hospital unit costs for each resource were calculated from hospitalized patients encounters in 2020 by multiplying charges at a specific hospital by the hospital’s department specific cost-to-charge ratio (CCR). Hospitals use the CCR method to compare costs between each other because comprehensive cost accounting is not available. Medication doses were expressed as one standard dose and not an entire treatment course.

Responses were summarized with frequencies and percentages and compared across categories using chi-square tests. The percent difference in estimated and CCR costs were calculated for each respondent and corresponding hospital. These were summarized using medians with inter-quartile ranges (IQR) and compared across groups using Wilcoxon Rank-Sum tests or Kruskal-Wallis tests as appropriate. All statistical analyses were performed in SAS v.9.4 (SAS Institute, Cart, NC) and p-values <0.05 were considered statistically significant.

## Results

Forty surveys completed by individuals who were not attendings, such as fellows, residents, and nurse practitioners, were excluded. Of the 49 hospitals, 40 (81.6%) had at least one response (range 1 to 41) with wide geographic representation. The total number of pediatric hospitalists at the 49 hospitals (1850) was determined by identifying pediatric hospitalist attendings listed on hospital or university websites. Of the 1850 attendings, 327 (17.7%) responses were received, including 270 (14.6%) complete responses. Most respondents identified as female and were 6–15 years into practice (Table 1).

**Table 1:** Demographics and self-reported cost-consciousness.

			To what degree did cost consciousness influence your practice?			
		Overall	Never/Rarely	Somewhat often	Very Often / Always	p
Gender						
	Male	99 (30.3)	16 (16.3)	43 (43.9)	39 (39.8)	0.01
	Female	226 (69.1)	52 (23.4)	113 (50.9)	57 (25.7)	
	Missing/Choose not to answer	2 (0.6)	2 (100)	0	0	
Years of practice						
	0–5 years	52 (15.9)	15 (29.4)	22 (43.1)	14 (27.5)	0.33
	6–15 years	206 (63)	43 (21.3)	103 (51)	56 (27.7)	
	16+ years	69 (21.1)	12 (17.4)	31 (44.9)	26 (37.7)	

### Self-reported cost consciousness

Most respondents considered cost into overall decision-making “somewhat often” (47.7%), which was also seen in each sub-category: laboratory studies (41.3%), medications (44.0%), and imaging studies (41.9%) (Table 2). Males self-reported higher degrees of cost consciousness than females (Table 1).

**Table 2:** Self-reported cost-consciousness.

	Never	Rarely	Somewhat often	Very often	Always
To what degree did cost consciousness influence your practice?					
	1 (0.3)	69 (21.1)	156 (47.7)	86 (26.3)	10 (3.1)
How often did you consider the cost of a laboratory study before ordering it?					
	7 (2.1)	90 (27.5)	135 (41.3)	83 (25.4)	7 (2.1)
How often did you consider the cost of a medication before ordering it?					
	8 (2.4)	115 (35.2)	144 (44)	49 (15)	6 (1.8)
How often did you consider the cost of an imaging study before ordering it?					
	5 (1.5)	97 (29.7)	137 (41.9)	73 (22.3)	10 (3.1)

### Self-reported knowledge of national medical waste reduction initiatives and hospital costs

While 66.9% of respondents had gained knowledge of national medical waste reduction initiatives through various avenues (including advanced degree coursework, lectures, and self-directed learning), 33.1% had no specific knowledge of those initiatives. Nearly all respondents (98.5%) felt that these initiatives should be introduced in either medical school (68.5%) or residency (30%). Those reporting that cost consciousness”Always” or “Very Often” influenced their practice were more likely to receive knowledge from self-directed learning compared to other groups (p = 0.02). Similarly, 24.3% of respondents reported having no specific knowledge of hospital costs with 96.5% supporting education about hospital costs during medical school (54%) or residency (42.5%) (Table 3).

**Table 3:** Self-reported knowledge of national medical waste reduction initiatives and hospital costs.

			To what degree did cost consciousness influence your practice?			
		Overall	Never / Rarely	Somewhat often	Very Often / Always	p
How did you obtain the largest percentage of knowledge regarding national waste reduction initiatives?						
	Advanced Degree (includes lecture/coursework as part of degree such as MBA/MHA)	14 (4.4)	5 (7.2)	6 (3.9)	3 (3.1)	0.02
	Attended course/lecture/informational session (QI project, Grand Rounds, Society Annual Meetings)	113 (35.6)	21 (30.4)	55 (36.2)	37 (38.5)	
	Knowledge accumulated while caring for a specific patient	26 (8.2)	3 (4.3)	15 (9.9)	8 (8.3)	
	Self-directed learning outside of caring for a specific patient	59 (18.6)	8 (11.6)	24 (15.8)	27 (28.1)	
	I have not obtained specific knowledge regarding national waste reduction initiatives	105 (33.1)	32 (46.4)	52 (34.2)	21 (21.9)	
When should trainees (medical students, residents, fellows) first be educated on national waste reduction initiatives?						
	During medical school	217 (68.5)	52 (75.4)	101 (66.4)	64 (66.7)	0.31
	During residency	95 (30)	17 (24.6)	46 (30.3)	32 (33.3)	
	During fellowship	0	0	0	0	
	As attendings	4 (1.3)	0	4 (2.6)	0	
	No formal education should be conducted (ie only via direct patient care or self-directed learning)	1 (0.3)	0	1 (0.7)	0	
How did you obtain the largest percentage of knowledge regarding your hospital’s costs for laboratory studies, medications, and imaging studies?						
	Advanced Degree (includes lecture/coursework as part of degree such as MBA/MHA)	8 (2.6)	3 (4.5)	2 (1.3)	3 (3.2)	0.28
	Attended course/lecture/informational session (QI project, Grand Rounds, Society Annual Meetings)	68 (21.7)	14 (20.9)	31 (20.5)	23 (24.2)	
	Knowledge accumulated while caring for a specific patient	80 (25.6)	12 (17.9)	45 (29.8)	23 (24.2)	
	Self-directed learning outside of caring for a specific patient	81 (25.9)	16 (23.9)	36 (23.8)	29 (30.5)	
	I have not obtained specific knowledge regarding hospital costs	76 (24.3)	22 (32.8)	37 (24.5)	17 (17.9)	
When should trainees (medical students, residents, fellows) first be educated on hospital costs?						
	During medical school	169 (54)	38 (56.7)	78 (51.7)	53 (55.8)	0.10
	During residency	133 (42.5)	25 (37.3)	66 (43.7)	42 (44.2)	
	During fellowship	2 (0.6)	2 (3)	0	0	
	As attendings	7 (2.2)	2 (3)	5 (3.3)	0	
	No formal education should be conducted (ie only via direct patient care or self-directed learning)	2 (0.6)	0	2 (1.3)	0	

### Cost estimates of commonly ordered laboratory studies, medications, and imaging studies

For each ordered item, the unit cost was determined by multiplying charges at a specific hospital by the hospital’s specific CCR, from which a median and IQR was calculated (Table 4).

**Table 4:** Median [interquartile range] unit cost and percent difference.

	Median [IQR] Unit cost	Median [IQR] Percent difference *
C-reactive protein	20.98 [12.04, 32.52]	153.7 [17.2, 441.1]
Basic Metabolic Panel	40.76 [18.82, 73.37]	30.6 [-43.8, 213.5]
Complete Blood Count with Differential	24.58 [14.24, 39.61]	103.4 [-23.4, 400]
Erythrocyte Sedimentation Rate	14.49 [6.39, 24.99]	284.7 [76.1, 803.4]
Aerobic Blood Culture	31.75 [14.73, 43.9]	202.1 [48.6, 718.8]
Amoxicillin 400mg per 5ml Oral	0.96 [0.22, 3.18]	657.5 [84, 4259]
Amoxicillin-Clavulanate acid 600mg per 5ml Oral	2.02 [0.44, 5.47]	787.2 [131.2, 3264.9]
Clindamycin 75mg per 5ml Oral	1.4 [0.45, 4.48]	934.5 [187.7, 3292.6]
Ampicillin 500mg Intravenous	9.41 [2.05, 15.12]	623 [115, 3321.4]
Clindamycin 150mg Intravenous	7.7 [1.22, 15.49]	931.2 [273.1, 3992.2]
Ceftriaxone 1000mg Intravenous	3.78 [0.87, 13.21]	1644.1 [296.5, 8582.9]
Ampicillin-Sulbactam 1500mg Intravenous	12.78 [3.06, 17.9]	795.3 [213.8, 3264.2]
Vancomycin 500mg Intravenous	13.67 [3.58, 26.03]	778.1 [160.7, 3448.8]
Chest X-ray (2-view)	105.35 [63.18, 137.24]	28.2 [-52.7, 136]
Abdominal X-ray (KUB)	77.04 [55.3, 116.31]	40.2 [-43.6, 184.8]
Renal Ultrasound	183.74 [133.69, 260.53]	18.7 [-55.5, 148.1]
Head Computerized Tomography (CT)	149.11 [76.56, 293.77]	150.8 [2.1, 539.7]
Echocardiogram Transthoracic	163.56 [99.9, 236.31]	295.5 [58.6, 691]
Brain Magnetic Resonance Imaging (MRI)	397.72 [188.46, 720.48]	131.7 [-24.2, 421.8]

* Percent difference of respondents’ cost estimate to hospital-specific unit cost

**Fig 1:**
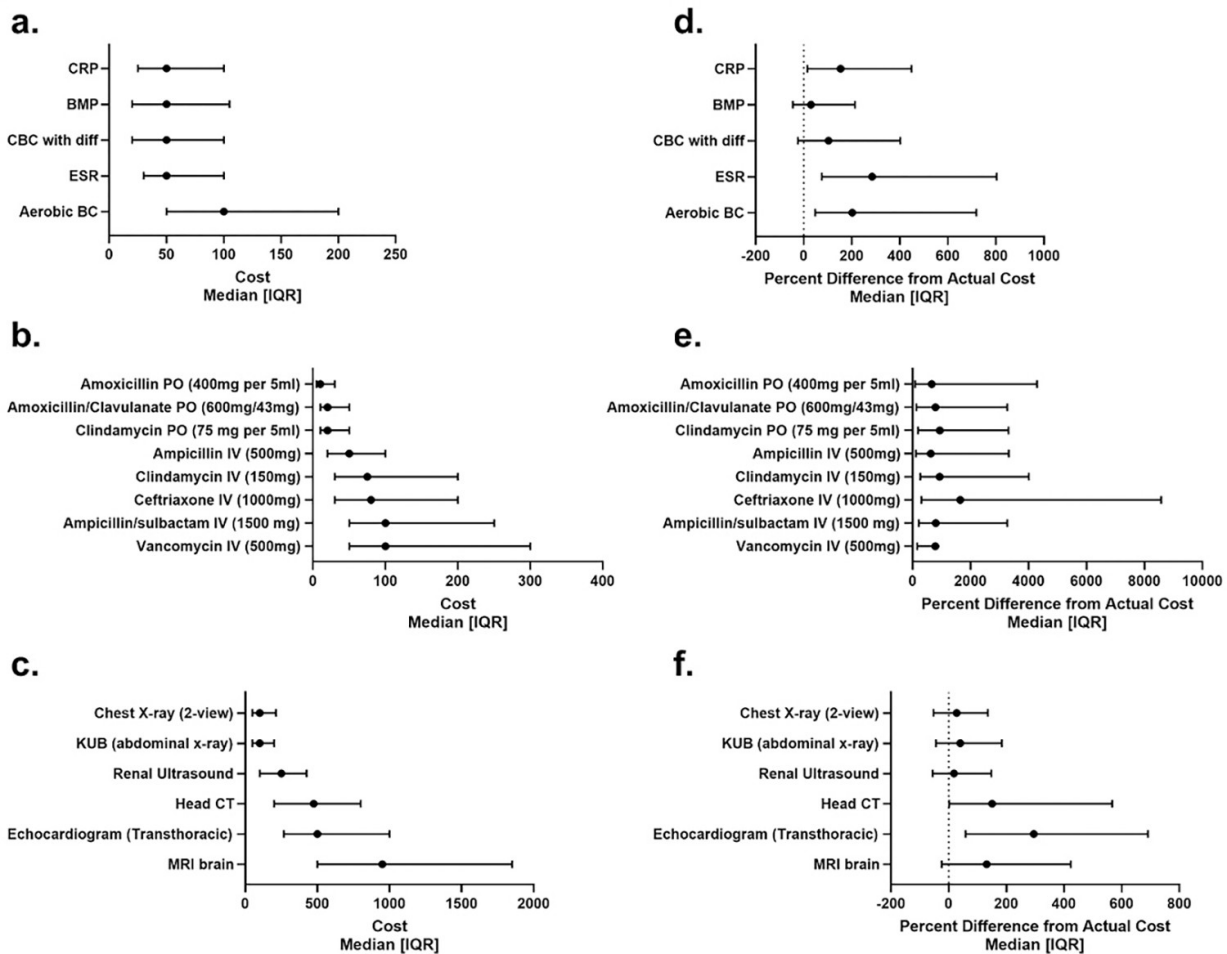
Median hospital-specific cost (1a-1c) and percent [interquartile] difference of respondent estimates from hospital cost of commonly ordered items (1d-1f). CRP = C-reactive protein, BMP = Basic metabolic profile, CBC = Complete blood count, ESR = Erythrocyte sedimentation rate, BC = Blood culture, PO = Oral, IV = Intravenous, mg = milligram, ml = milliliter, CT = Computer tomography, MRI = Magnetic resonance imaging.

Respondents’ cost estimates were compared to hospital-specific costs and displayed as median and IQR percent difference from the actual cost (Table 4). Medications were lower cost and had the largest median percent difference compared to laboratory and imaging studies, which had higher costs and smaller percent differences (Fig 1). The median percent difference was above 0% (range 1.4–1644.1%), meaning an overestimation of cost for all resources. The item with the greatest percent difference was intravenous ceftriaxone, which was estimated at 16 times higher than the CCR cost (Table 4). Attendings with high levels of self-reported cost-consciousness (considering cost “Always” or “Very Often” in their decision-making) tended to overestimate costs with a median percent difference of 234.1% vs. 124.1% for laboratory studies (p = <0.01), 1094.7% vs. 735.2% for medications (p = 0.01), and 131.9% vs. 79.4% for imaging studies (p = 0.04; Table 5) when compared to the “Somewhat Often”, “Never”, and “Rarely” groups. Compared to respondents with <16 years of practice, those with 16+ years in practice had more accurate cost estimations for medications (457.9% vs 908.6%, p<0.01) and imaging studies (76.5% vs. 102.5%, p<0.01), but no difference for laboratory studies (Table 6).

**Table 5:** Median [interquartile range] percent difference and self-reported cost consciousness.

	Median [IQR] percent difference *	Median [IQR] percent difference To what degree did cost consciousness influence your practice?			
	Overall	Never/Rarely	Somewhat often	Very Often / Always	p
**Laboratory Studies**					
C-reactive protein	153.7 [17.2, 441.1]	120.4 [-21.9, 328.7]	149.9 [24.9, 407.4]	243.4 [52.4, 830.5]	0.01
Basic Metabolic Panel	30.6 [-43.8, 213.5]	-5.1 [-68.7, 142.2]	20.8 [-41.5, 184.2]	93.8 [-33.9, 325.4]	0.03
Complete Blood Count with Differential	103.4 [-23.4, 400]	74.1 [-52.8, 342.3]	89.3 [-15.2, 341.8]	251 [-8.3, 608.6]	<0.01
Erythrocyte Sedimentation Rate	284.7 [76.1, 803.4]	177 [5, 577.5]	284.7 [74.8, 668.5]	372.3 [95.9, 1251.8]	0.04
Blood Culture	202.1 [48.6, 718.8]	195.9 [20.6, 511.8]	202.1 [51.1, 614.1]	382.9 [48.6, 791.6]	0.30
**Medications**					
Amoxicillin 400mg per 5ml Oral	657.5 [84, 4259]	647.5 [49.5, 4624.4]	667.5 [86.9, 2490.3]	677.1 [83.5, 6522.7]	0.57
Amoxicillin-Clavulanate acid 600mg per 5ml Oral	787.2 [131.2, 3264.9]	787.3 [77.4, 3240.5]	729.8 [131.4, 2890.8]	1029.1 [199.1, 6629.8]	0.48
Clindamycin 75mg per 5ml Oral	934.5 [187.7, 3292.6]	890.2 [126.9, 3540.9]	642.5 [183.6, 3198.6]	1338.5 [256.9, 5987.4]	0.12
Ampicillin 500mg IV	623 [115, 3321.4]	603.6 [145.1, 3473.5]	455.9 [61.2, 3230.2]	700 [145.1, 3321.4]	0.55
Clindamycin 150mg IV	931.2 [273.1, 3992.2]	816.6 [141, 4803.3]	757.4 [224.7, 3631.3]	1180.5 [340.2, 6302.3]	0.29
Ceftriaxone 1000mg IV	1644.1 [296.5, 8582.9]	2654.4 [590.8, 11110.3]	1515.4 [255.4, 8582.9]	1745.7 [263.6, 7601.1]	0.27
Ampicillin-Sulbactam 1500mg IV	795.3 [213.8, 3264.2]	909.6 [257.2, 3676.9]	741.1 [150.7, 3264.2]	1155.1 [324.5, 2927.5]	0.57
Vancomycin 500mg IV	778.1 [160.7, 3448.8]	798.9 [191.3, 4387]	566.4 [101.6, 3231.9]	1203.6 [224, 3741.4]	0.33
**Imaging Studies**					
Chest X-ray (2-view)	28.2 [-52.7, 136]	28.3 [-58.5, 113.8]	6.9 [-51.4, 119.6]	43.5 [-52.6, 291.4]	0.24
Abdominal X-ray (KUB)	40.2 [-43.6, 184.8]	38.5 [-55.1, 181.2]	34.6 [-43.1, 169.2]	80.9 [-49.3, 289.3]	0.45
Renal Ultrasound	18.7 [-55.5, 148.1]	76.5 [-31.7, 143.7]	6.1 [-57.6, 145.1]	11.9 [-56.5, 151.4]	0.89
Head Computerized Tomography (CT)	150.8 [2.1, 539.7]	183.8 [-4.2, 443.5]	123.6 [4.1, 539.7]	169.4 [-1.1, 662.8]	0.86
Echocardiogram Transthoracic	295.5 [58.6, 691]	340.5 [62.6, 691]	255.3 [58.2, 662.7]	317.6 [60.2, 858.3]	0.46
Brain Magnetic Resonance Imaging (MRI)	131.7 [-24.2, 421.8]	112.2 [-24.2, 393.7]	127.3 [-24.2, 374.4]	192.3 [-14.5, 490.6]	0.56
**Categories**					
Laboratory Studies	146.8 [-2.9, 506]	103.9 [-33.2, 353.2]	129.2 [3.1, 455.4]	234.1 [33.6, 735.9]	<0.01
Medications	826.2 [176.8, 3812.3]	882.1 [155.7, 4509.9]	697.6 [149.4, 3264.9]	1094.7 [227.5, 5146.1]	0.01
Imaging Studies	91.2 [-27.4, 367.9]	104.3 [-24.2, 329.9]	76.5 [-28.7, 327.3]	131.9 [-22.8, 460.7]	0.12

* Percent difference of respondents’ cost estimate to hospital-specific unit cost

**Table 6:** Median [interquartile range] percent difference and years of practice.

		Years of practice			
	Overall *	0–5 years	6–15 years	16+ years	p
Categories					
Laboratory Studies	146.8 [-2.9, 506]	139.2 [-5.9, 454.3]	141.1 [-5.9, 494.4]	183.1 [11.9, 599.4]	0.34
Medications	826.2 [176.8, 3812.3]	1673 [526.3, 6463.1]	744.2 [153.5, 3445.6]	457.9 [85.2, 3292.6]	<0.01
Imaging Studies	91.2 [-27.4, 367.9]	197.1 [27.3, 534.6]	82.3 [-28.7, 327.1]	76.5 [-34.1, 374.4]	<0.01

* Percent difference of respondents’ cost estimate to hospital-specific unit cost

## Discussion

In this national survey of pediatric hospitalists, one-third of respondents had no specific knowledge of national medical waste reduction initiatives and nearly one-quarter were unaware of hospital specific costs. Respondents overestimated the cost of every item and those who self-reported high cost-consciousness were generally the least cost accurate (largest difference between estimate and hospital CCR cost). Respondents with 16+ years of practice were the most cost accurate (smallest difference between estimate and hospital CCR cost). These findings suggest that knowledge of national initiatives may not be adequate to inform physicians about the costs of health care and that attending physician cost accuracy may not equate to lower health care costs.

### National medical waste reduction initiatives and hospital-specific costs

National medical waste reduction initiatives were implemented to help reduce health care costs in the United States by reducing overutilization. In our study, those who self-reported knowledge of initiatives tended to be more cost-conscious, and those who self-reported no knowledge of initiatives tended to be less cost-conscious, however, high self-reported cost-consciousness did not lead to higher cost accuracy. National medical waste reduction initiatives focus on eliminating unnecessary interventions without specifically mentioning the cost of those interventions. Thus, one can consider abstract cost reduction during medical practice without knowledge of hospital-specific costs. Previous literature suggests that passive awareness of national medical waste reduction initiatives alone does not lead to durable change in physicians’ ordering behavior [21]. Other targeted interventions such as clinical pathways [22], best practice alerts [23], or cost display [24] in the electronic health record could reduce health care cost better rather than solely focusing on improving cost accuracy.

The lack of cost transparency may be a contributing factor to the 24.3% of respondents who continue to be unaware of local hospital-specific costs of commonly ordered laboratory studies, medications, and imaging studies. Although hospital chargemaster sheets are available, the true cost of an item is a fraction of the charge and varies from hospital to hospital, thus we used CCR cost for interhospital comparisons. A recent Presidential Executive Order, which would require health plans to provide the negotiated rates and out-of-pocket costs to consumers in 2023, may aid in cost transparency and physician awareness of costs [25]. Prior cost transparency studies that focused on pilot programs aimed at reducing overutilization of specific items, reported conflicting outcomes [26–28]. Though some showed no changes or only short-term changes in behavior, programs with longer-term behavior change continued to stress the intervention over longer periods of time. Future studies could investigate the ability to change ordering behavior across multiple fields rather than one specific area.

Nearly all respondents support early introduction of hospital-specific costs and education on national medical waste reduction Initiatives during medical school or residency, a finding that supports previous calls to introduce these topics earlier in training [15, 24, 29]. Previous studies have shown an association between higher cost consciousness and longer clinical work experience [14], but studies have not looked at cost accuracy in relation to clinical work experience. Late career respondents (16+ years of practice) were statistically more accurate in the medications and imaging studies categories compared to those with less than 16 years of practice (Table 6).

### Cost-consciousness versus cost-accuracy

Physicians, regardless of specialty, are not accurate with their estimations of costs for commonly ordered items [10–13]. These studies were primarily at single institutions with only one focused on general pediatricians [10]. Our study is unique as it is the first national survey of pediatric hospitalists that examines the relationships between demographic data, cost-consciousness, and cost accuracy. Respondents reporting that cost consciousness “Always” of “Very Often” influenced their practice were more likely to have gained knowledge of national medical waste reduction initiatives from self-directed learning, outside of caring for a specific patient compared to other groups (p = 0.02, Table 3). In the overall group, over half (51.5%) obtained their knowledge outside an advanced degree or formal course, suggesting that most pediatric hospitalists learn about hospital costs on their own. This suggests that formal degrees or structured lectures may not be the most effective way to increase awareness of hospital-specific costs.

As a group, pediatric hospitalists overestimated the cost of every item in the survey, as shown by the median unit costs and percent differences (Table 4). This finding may explain why cost displays alone may not be effective [27]. When seeing that the cost display is lower than the pediatric hospitalist’s cost estimation, it may negatively affect the physician’s behavior since that item may be viewed as “less expensive” than originally thought and thus increase utilization. Further study would be needed to determine causation.

Medications had the lowest median unit cost compared to laboratory or imaging studies. The selected medications were low-cost items and inaccurate cost assignments to them led to larger percent differences (Table 4). Respondents with high levels of self-reported cost consciousness were not the most accurate, and in many instances, they had the highest percent difference (estimated cost from hospital-specific unit cost) compared to less cost-conscious groups (Table 5). Respondents with the lowest self-reported cost consciousness were statistically the most accurate regarding the cost of a complete blood count, basic metabolic panel, erythrocyte sedimentation rate and C-reactive protein. This suggests that self-perception of cost-consciousness does not translate to accurate cost estimations. Lastly, if hospital specific costs are unavailable to physicians, having national median unit costs (Table 4) may provide anchoring points for cost education, particularly for trainees and early career physicians.

### Limitations

Several limitations were noted. First, survey responses were limited. Tactics were used to improve the survey response rate: clear subject line, sent from someone the respondent knows (division directors), direct link to survey, and reminder every 2 weeks [30]. We relied on division directors to distribute the survey to the appropriate individuals, which may explain why some hospitals had no responses. There is no universal contact sheet, including organizational listservs, of pediatric hospitalists. While hospital and university websites might not be current, the listed faculty were counted and led to the denominator of 1850. Our response rate might not allow findings to be generalized to all pediatric hospitals or even within some individual hospitals. Second, the general link was not user specific, thus a respondent could have completed multiple surveys. Third, not every survey was complete and survey takers may have confused cost with charge or assigned a cost for a total course of medication rather than single dose, despite survey instructions. Fourth, cost consciousness was not explicitly defined in the survey, which opens it to respondent interpretation, but that question was grouped with other questions reflecting on cost when ordering specific items. Fifth, we selected commonly ordered laboratory studies, medications, and imaging studies, so our findings may not be generalizable to other items in those categories, particularly very high-cost items. Lastly, clinical outcomes and appropriateness of ordered items were not examined.

## Conclusions and future areas of research

We found no significant relationship between knowledge of national medical waste reduction initiatives and cost accuracy, which is not necessarily unexpected as national medical waste reduction initiatives are not based on knowing costs since any reduction of unnecessary interventions would reduce health care spending. Surprisingly, pediatric hospitalists overestimated cost across all domains which may lead to increased health care spending if the intervention was viewed as less expensive than originally thought. This finding may divert future studies away from cost accuracy of individual items as a way to reduce health care spending and instead focus on best practice guidelines or relative cost between similar items such as antibiotics. National median unit cost lists can be used in lieu of hospital specific cost sheets, in order to provide pediatric hospitalists the data needed to train residents and medical students about hospital costs. There remains a discordance between self-reported cost consciousness and cost accuracy; high cost-conscious respondents were the least accurate in estimating costs, which speaks to a general notion of cost rather than specific costs. Additional studies could explore other associations that translate to lasting, quantifiable reductions in health care costs and positive clinical outcomes, particularly in hospitalized pediatric patients.

## Supporting information

S1 File Cost conscioussness survey. PDF of the on-line survey.
(PDF)
