# Supplementary material for: National cross-sectional study on cost consciousness, cost accuracy, and national medical waste reduction initiative knowledge among pediatric hospitalists in the United States
Source: PLoS One. 2023 Apr 24;18(4):e0284912. doi: 10.1371/journal.pone.0284912 (PMC10124847; doi:10.1371/journal.pone.0284912)
Supplement: S1 File — PDF of the on-line survey. (PDF) [file pone.0284912.s001.pdf]

Cost Consciousness Survey

In 2018, the United States spent 18% of its gross domestic product ($3.76 trillion) on health care. Studies estimate that 30% of health care spending is waste.

National waste reduction initiatives such as Choosing Wisely®, Pediatric Respiratory Illness Measurement System (PRIMES), and Agency for Healthcare Research and Quality- Pediatric Quality Measures Program (AHRQ-PQMP), aim to reduce unnecessary laboratory testing, medications and imaging studies.

Physicians have variable accuracy regarding the costs of commonly ordered laboratory tests, medications, and imaging studies. Little is known regarding physicans' cost consciousness and their knowledge and use of national waste reduction initiatives, particularly at pediatric hospitals.

The aims of this 7-section survey are to

1) Identify physician awareness and use of national medical waste initiatives

2) Determine physicians' accuracy reagrding the cost of commonly ordered laboratory studies, medications, and imaging studies at their specific hospital

Section 1 Demographics

Gender

- ◯ Male
- ◯ Female
- ◯ Non-Binary
- ◯ Choose not to answer

Years of Practice Including Residency and Fellowship

- ◯ 0-5 years
- ◯ 6-15 years
- ◯ 16+ years

Current Year of Training

- ◯ PGY-1 to PGY-3
- ◯ PGY-4 to PGY-6+
- ◯ Attending
- ◯ Other (Nurse Practitioner, Physician Assistant, Clinical Nurse Specialist, etc)

City and Hospital Where You Spend Most of Your Clinical Time

- ◯ Akron: Akron CH
- ◯ Akron: Akron General Medical Center (Neonatal Unit)
- ◯ Akron: Mahoning Valley
- ◯ Akron: St. Elizabeth
- ◯ Ann Arbor: Ann Arbor CS Mott Children
- ◯ Atlanta: Egleston
- ◯ Atlanta: Scottish Rite
- ◯ Austin: Austin Dell CMC
- ◯ Birmingham: Birmingham Child Alabama
- ◯ Boston: Boston CH
- ◯ Charleston: Charleston CH MUSC
- ◯ Charlotte: Jeff Gordon Children's Hospital
- ◯ Charlotte: Levine Children's Hospital
- ◯ Chicago - Advocate: Oak Lawn Advocate CH
- ◯ Chicago - Advocate: Park Ridge Advocate CH
- ◯ Chicago - Comer: Comer
- ◯ Chicago - Comer: Mitchell
- ◯ Chicago - Lurie: Chicago Lurie CH
- ◯ Cincinnati: Cincinnati CH
- ◯ Cleveland: Rainbow UH Ahuja Medical Center
- ◯ Cleveland: Rainbow UH Case Medical Center
- ◯ Cleveland: Rainbow UH St. John Medical Center
- ◯ Columbus: Columbus Nationwide CH
- ◯ Dallas: Children's Medical Center Dallas
- ◯ Dallas: Children's Medical Center Legacy
- ◯ Dallas: Our Children's House
- ◯ Denver: Children's Hospital Colorado at Memorial Main Campus
- ◯ Denver: Children's Hospital Colorado South Campus
- ◯ Denver: Children's Hospital Colorado Main Campus
- ◯ Fort Worth: Cook Children's Health Care System
- ◯ Hartford: Hartford Connecticut CMC
- ◯ Houston: Main
- ◯ Houston: West
- ◯ Houston: Women's Pavilion
- ◯ Houston: Woodlands
- ◯ Houston - Hermann: Children's Hospital Memorial Hermann
- ◯ Indianapolis: Indianapolis Riley
- ◯ Indianapolis: University
- ◯ Kansas City: Children's Mercy Hospital Kansas
- ◯ Kansas City: Children's Mercy Hospitals and Clinics Main Campus
- ◯ Little Rock: Arkansas Children's Hospital
- ◯ Little Rock: Arkansas Childrens NW
- ◯ Loma Linda: Loma Linda Univ CH
- ◯ Long Beach: Long Beach Miller CH
- ◯ Los Angeles: Los Angeles CH
- ◯ Louisville: KCH
- ◯ Louisville: Norton St Matthews
- ◯ Madera: Madera Valley CH
- ◯ Memphis - Le Bonheur: Le Bonheur Children's Hospital - Inpatient
- ◯ Miami: Nicklaus CH
- ◯ Milwaukee: Children's Hospital of Wisconsin
- ◯ Milwaukee: Fox Valley
- ◯ Minneapolis - St. Paul: Children's Hospitals and Clinics of Minnesota: Minneapolis
- ◯ Minneapolis - St. Paul: Children's Hospitals and Clinics of Minnesota: St. Paul
- ◯ Nashville: Monroe Carrell
- ◯ New Haven: New Haven Yale CH
- ◯ New Haven: Yale-New Haven Children's Hospital
- ◯ New York: Presbyterian Komansky Center for Children's Health
- ◯ New York: Presbyterian Morgan Stanley Children's Hospital
- ◯ New York: Presbyterian Lower Manhattan

◯ New York: Presbyterian-Sloane Hospital for Women
◯ Norfolk: Norfolk CHKD
◯ Oakland: Oakland CH
◯ Omaha: Omaha CH
◯ Orange: Orange CHOC
◯ Palo Alto: Palo Alto Lucile Packard
◯ Philadelphia: Philadelphia CHOP
◯ Phoenix: Phoenix CH
◯ Pittsburgh: Main Hospital
◯ Salt Lake City: PCMC Riverton Hospital Outpatient Center
◯ Salt Lake City: Primary Children's Medical Center
◯ San Diego: San Diego Rady CH
◯ Seattle: Seattle Childrens
◯ St. Louis: St Louis CH
◯ St. Petersburg: All Children's Hospital
◯ Washington DC: Washington CNMC
◯ Not Listed

Please enter your (City: Hospital Name) ________________________________

Section 2 Reflect on a typical week of service

	Never	Rarely	Somewhat often	Very often	Always
To what degree did cost consciousness influence your practice?	◯	◯	◯	◯	◯
How often did you consider the cost of a laboratory study before ordering it?	◯	◯	◯	◯	◯
How often did you consider the cost of a medication before ordering it?	◯	◯	◯	◯	◯
How often did you consider the cost of an imaging study before ordering it?	◯	◯	◯	◯	◯

Section 3 Education on national waste reduction initiatives
Examples include: Choosing Wisely®, Pediatric Respiratory Illness Measurement System (PRIMES), and Agency for Healthcare Research and Quality- Pediatric Quality Measures Program (AHRQ-PQMP)

How did you obtain the largest percentage of knowledge regarding national waste reduction initiatives?

- ◯ Advanced Degree (includes lecture/coursework as part of degree such as MBA/MHA)
- ◯ Attended course/lecture/informational session (QI project, Grand Rounds, Society Annual Meetings)
- ◯ Knowledge accumulated while caring for a specific patient
- ◯ Self-directed learning outside of caring for a specific patient
- ◯ I have not obtained specific knowledge regarding national waste reduction initiatives

When should trainees (medical students, residents, fellows) first be educated on national waste reduction initiatives?

- ◯ During medical school
- ◯ During residency
- ◯ During fellowship
- ◯ As attendings
- ◯ No formal education should be conducted (ie only via direct patient care or self-directed learning)

Section 4 Education on hospital costs

Cost = the number of US dollars the hospital pays for the item

How did you obtain the largest percentage of knowledge regarding your hospital's costs for laboratory studies, medications, and imaging studies?

- ◯ Advanced Degree (includes lecture/coursework as part of degree such as MBA/MHA)
- ◯ Attended course/lecture/informational session (QI project, Grand Rounds, Society Annual Meetings)
- ◯ Knowledge accumulated while caring for a specific patient
- ◯ Self-directed learning outside of caring for a specific patient
- ◯ I have not obtained specific knowledge regarding hospital costs

When should trainees (medical students, residents, fellows) first be educated on hospital costs?

- ◯ During medical school
- ◯ During residency
- ◯ During fellowship
- ◯ As attendings
- ◯ No formal education should be conducted (ie only via direct patient care or self-directed learning)

Section 5 Knowledge of laboratory costs

Cost = the number of US dollars the hospital pays for the item
Charge = the number of US dollars the hospital bills a patient/insurance company for the item
Generally, the cost is a fraction of the charge.

Please assign the cost (in dollars) for each of the following laboratory studies at your hospital.

Even if you are unsure, enter your best estimate.

Complete Blood Count with differential (CBC with diff) ________________________

Basic Metabolic Panel (BMP) ________________________

Respiratory Viral Panel (RVP) ________________________

Blood culture (aerobic) ________________________

Erythrocyte Sedimentation Rate (ESR) ________________________

C-Reactive Protein (CRP) ________________________

Section 6 Knowledge of medication costs

Cost = the number of US dollars the hospital pays for the item
Charge = the number of US dollars the hospital bills a patient/insurance company for the item
Generally, the cost is a fraction of the charge.

Please assign the cost (in dollars) for each of the following medications (dose) at your hospital.
Even if you are unsure, enter your best estimate.

Ampicillin IV (500 mg)	__________________
Ampicillin/sulbactam IV (1500 mg)	__________________
Amoxicillin PO suspension (400mg per 5ml)	__________________
Amoxicillin/Clavulanate suspension PO (600mg/43mg per 5ml)	__________________
Ceftriaxone IV (1000 mg)	__________________
Clindamycin IV (150 mg)	__________________
Clindamycin PO suspension (75 mg per 5ml)	__________________
Vancomycin IV (500 mg)	__________________

Section 7 Knowledge of imaging study costs

Cost = the number of US dollars the hospital pays for the item
Charge = the number of US dollars the hospital bills a patient/insurance company for the item
Generally, the cost is a fraction of the charge.

Please assign the cost (in dollars) for each of the following imaging studies at your hospital.
This does not include the radiology reading fee.
Even if you are unsure, enter your best estimate.

Chest X-ray (2-view) __________________________________

KUB (abdominal x-ray) __________________________________

Renal Ultrasound __________________________________

Head CT __________________________________

MRI brain __________________________________

Echocardiogram (Transthoracic) __________________________________
